# Supplementary figures and images for: Convective forces contribute to post‐traumatic degeneration after spinal cord injury
Source: Bioeng Transl Med. 2025 Jan 14;10(2):e10739. doi: 10.1002/btm2.10739 (PMC11883127; doi:10.1002/btm2.10739)

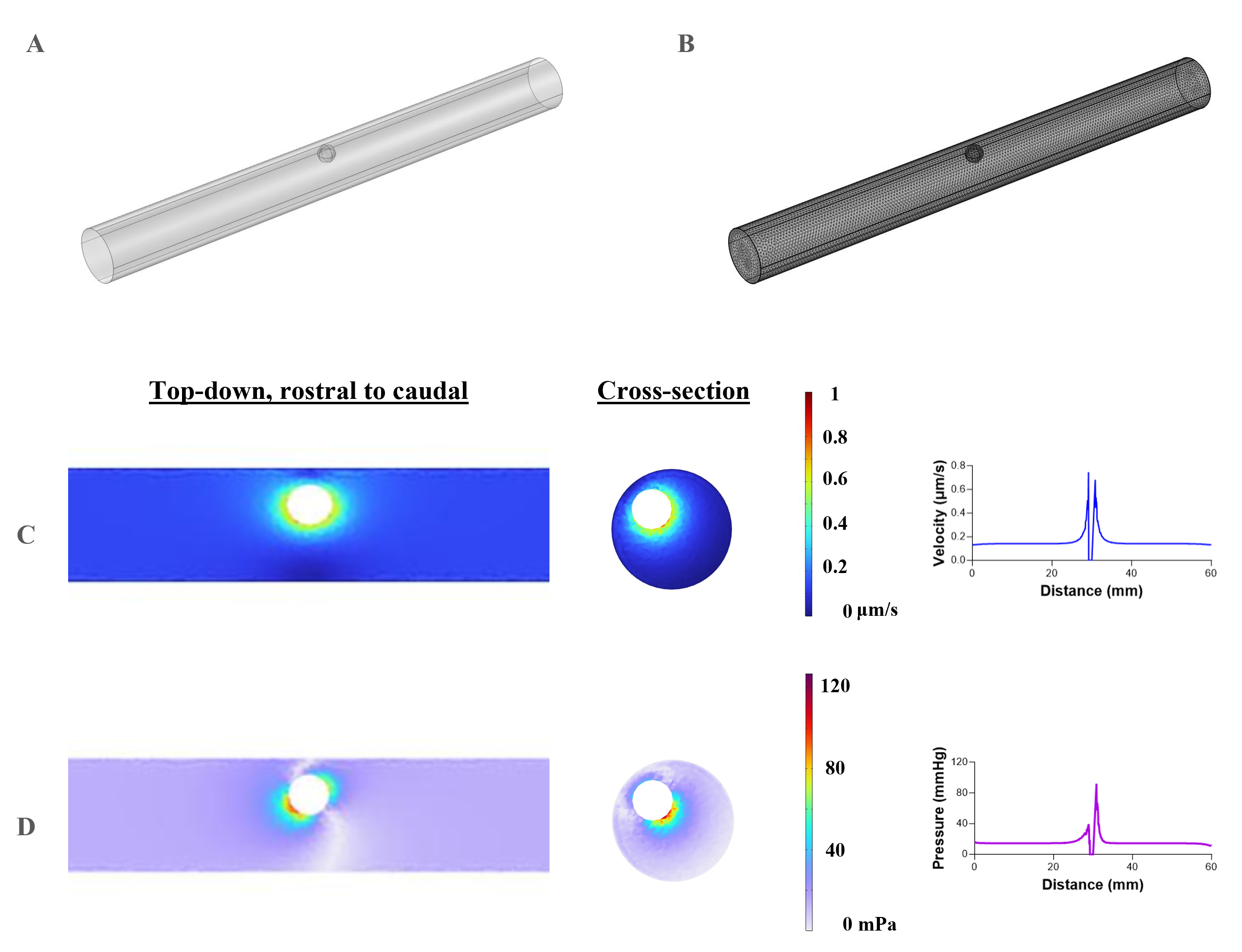

Supplement: Supplementary file 1 — FIGURE S1. (a) Representation of the COMSOL model. (b) Mesh generated with finer setting. 593,886 domain elements, 21,320 boundary elements, and 824 edge elements. (c,d) Velocity (c) and shear stress (d) of 3 DPI model without incoming interstitial fluid flow in the +x direction. The peak magnitude is higher compared to the IFF model, and the flow is isotropic in the x direction. [file BTM2-10-e10739-s001.png]

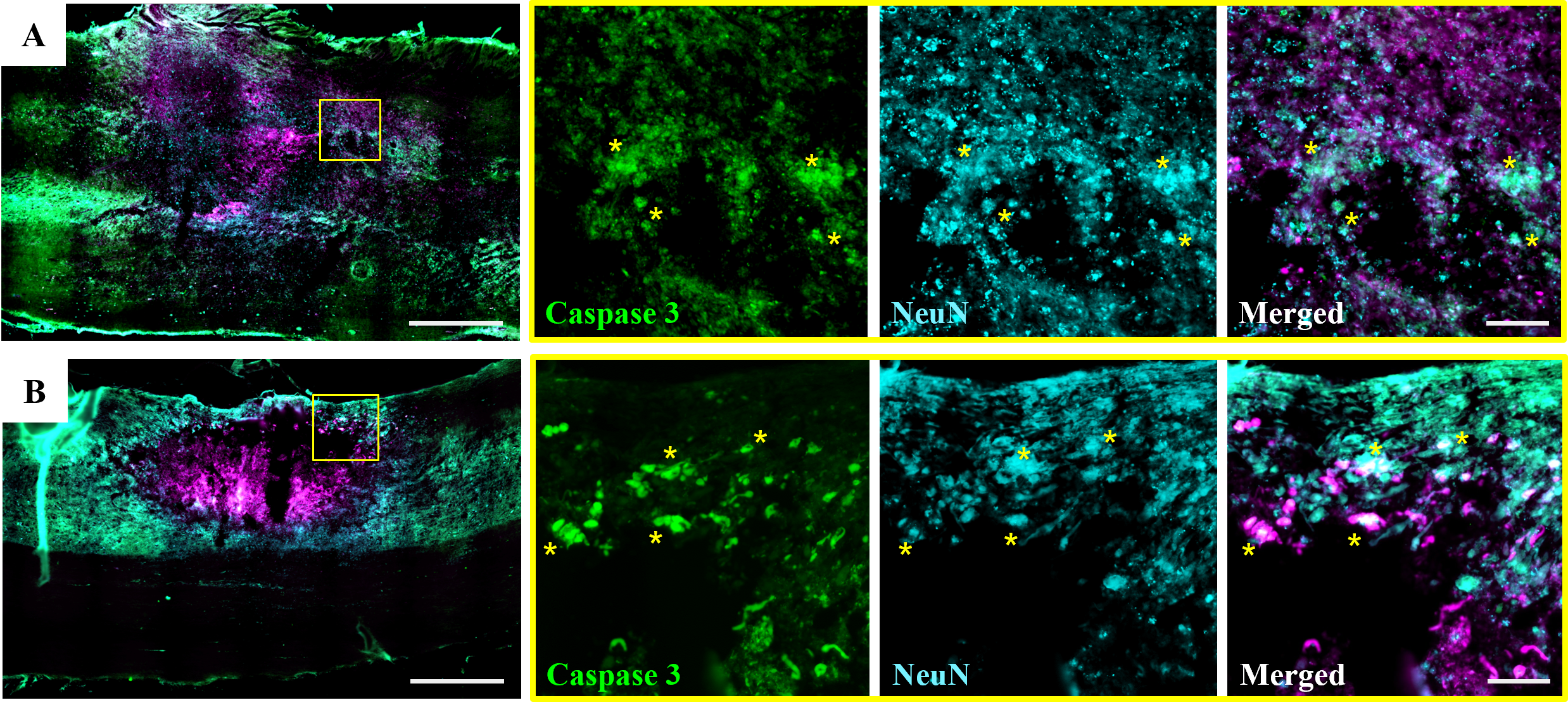

Supplement: Supplementary file 2 — FIGURE S2. Representative fluorescence images of spinal cord sections at 3 DPI (a) and 7 DPI (b) co‐labeled for cleaved caspase‐3 (green), neuronal nuclei (NeuN, cyan), and Evans Blue (magenta). Inset images show a higher magnification of the region outline by the yellow box, with stars indicating example caspase+NeuN+ cells, or apoptotic neurons. Scale bars are 1 mm for images on the left, and 100 μm for the inset images. [file BTM2-10-e10739-s003.png]

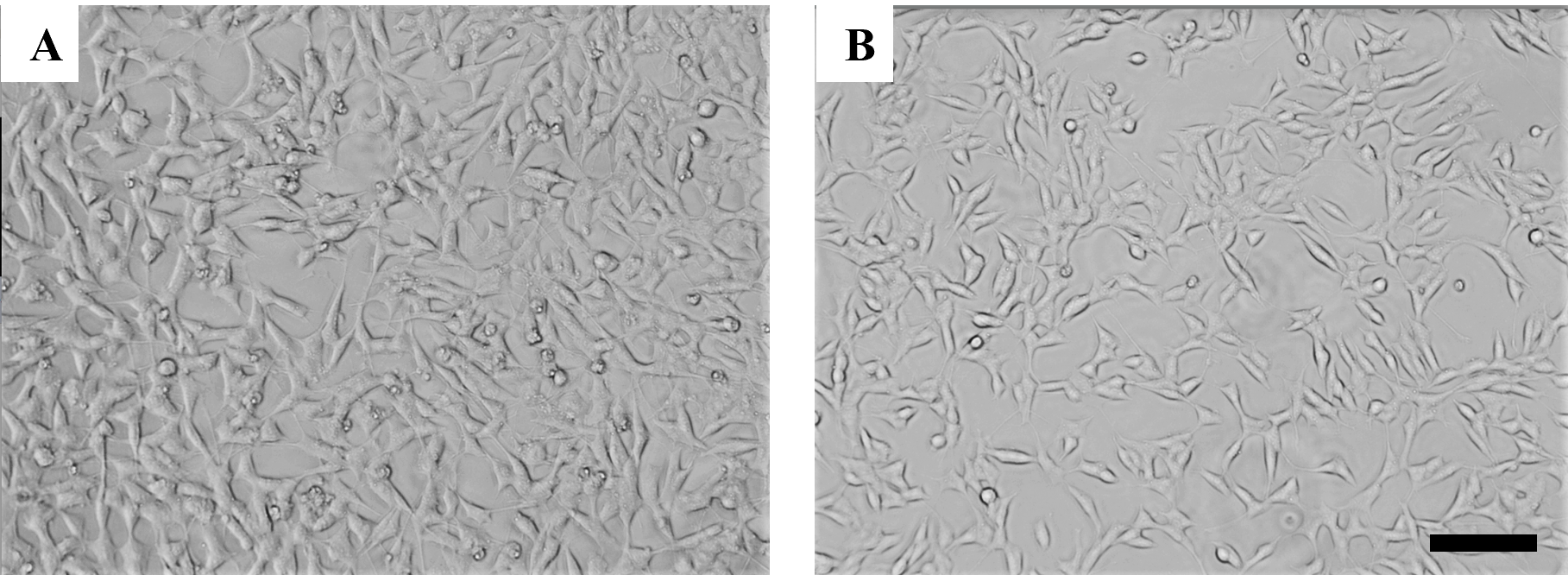

Supplement: Supplementary file 3 — FIGURE S3. (a) Representative transmitted light image of SH‐SY5Y neuronal‐like cells prior to the start of shear. (b) Representative transmitted light image of SH‐SY5Y neuronal‐like cells after overnight shear. Scale bar is 50 μm. [file BTM2-10-e10739-s002.png]
